# Supplementary material for: Modeling glioblastoma heterogeneity as a dynamic network of cell states
Source: Mol Syst Biol. 2021 Sep 16;17(9):e10105. doi: 10.15252/msb.202010105 (PMC8444284; doi:10.15252/msb.202010105)
Supplement: Supplementary file 6 — Source Data for Figure 5 [file MSB-17-e10105-s004.zip › Figure5A_sourcedata/GSEA_3071/hallmarks_stateIV.GseaPreranked.1623742551702/HALLMARK_UNFOLDED_PROTEIN_RESPONSE.html]

Details for gene set HALLMARK\_UNFOLDED\_PROTEIN\_RESPONSE[GSEA]

|  || Dataset | state53071 |
| Phenotype | NoPhenotypeAvailable |
| Upregulated in class | na\_neg |
| GeneSet | HALLMARK\_UNFOLDED\_PROTEIN\_RESPONSE |
| Enrichment Score (ES) | -0.4293028 |
| Normalized Enrichment Score (NES) | -1.9936813 |
| Nominal p-value | 0.0 |
| FDR q-value | 0.0017400043 |
| FWER p-Value | 0.018 |
Table: GSEA Results Summary

  

Fig 1: Enrichment plot: HALLMARK\_UNFOLDED\_PROTEIN\_RESPONSE      
 Profile of the Running ES Score & Positions of GeneSet Members on the Rank Ordered List

  

| PROBE | GENE SYMBOL | GENE\_TITLE | RANK IN GENE LIST | RANK METRIC SCORE | RUNNING ES | CORE ENRICHMENT || 1 | EIF4A2 |  |  | 860 | 0.575 | -0.1466 | No |
| 2 | STC2 |  |  | 972 | 0.535 | -0.1513 | No |
| 3 | VEGFA |  |  | 1010 | 0.518 | -0.1425 | No |
| 4 | DCP1A |  |  | 1036 | 0.509 | -0.1316 | No |
| 5 | TUBB2A |  |  | 1373 | 0.422 | -0.1827 | No |
| 6 | SPCS1 |  |  | 1679 | 0.353 | -0.2301 | No |
| 7 | CNOT4 |  |  | 1804 | 0.330 | -0.2436 | No |
| 8 | CNOT2 |  |  | 2256 | 0.261 | -0.3218 | No |
| 9 | CNOT6 |  |  | 2291 | 0.257 | -0.3204 | No |
| 10 | DNAJC3 |  |  | 2334 | -0.251 | -0.3207 | No |
| 11 | SLC1A4 |  |  | 2350 | -0.252 | -0.3158 | No |
| 12 | KIF5B |  |  | 2471 | -0.266 | -0.3305 | No |
| 13 | BAG3 |  |  | 2472 | -0.267 | -0.3223 | No |
| 14 | SDAD1 |  |  | 2614 | -0.284 | -0.3405 | No |
| 15 | WIPI1 |  |  | 2738 | -0.298 | -0.3548 | No |
| 16 | SLC30A5 |  |  | 2844 | -0.312 | -0.3653 | No |
| 17 | BANF1 |  |  | 2865 | -0.315 | -0.3594 | No |
| 18 | ATF3 |  |  | 2977 | -0.330 | -0.3705 | No |
| 19 | EIF4A3 |  |  | 3116 | -0.349 | -0.3861 | No |
| 20 | EXOSC2 |  |  | 3268 | -0.370 | -0.4036 | No |
| 21 | EXOC2 |  |  | 3379 | -0.387 | -0.4127 | No |
| 22 | HERPUD1 |  |  | 3467 | -0.402 | -0.4169 | Yes |
| 23 | PREB |  |  | 3473 | -0.404 | -0.4055 | Yes |
| 24 | EXOSC1 |  |  | 3501 | -0.410 | -0.3980 | Yes |
| 25 | HSPA5 |  |  | 3555 | -0.419 | -0.3952 | Yes |
| 26 | IMP3 |  |  | 3626 | -0.433 | -0.3953 | Yes |
| 27 | NOLC1 |  |  | 3710 | -0.448 | -0.3974 | Yes |
| 28 | YIF1A |  |  | 3723 | -0.449 | -0.3859 | Yes |
| 29 | EXOSC10 |  |  | 3738 | -0.452 | -0.3746 | Yes |
| 30 | NOP14 |  |  | 3787 | -0.463 | -0.3696 | Yes |
| 31 | SEC31A |  |  | 3959 | -0.501 | -0.3868 | Yes |
| 32 | EIF4A1 |  |  | 4013 | -0.516 | -0.3811 | Yes |
| 33 | EXOSC9 |  |  | 4189 | -0.564 | -0.3972 | Yes |
| 34 | FKBP14 |  |  | 4240 | -0.576 | -0.3890 | Yes |
| 35 | HSPA9 |  |  | 4410 | -0.639 | -0.4016 | Yes |
| 36 | ERO1A |  |  | 4434 | -0.647 | -0.3861 | Yes |
| 37 | ATF4 |  |  | 4453 | -0.655 | -0.3694 | Yes |
| 38 | EIF4EBP1 |  |  | 4474 | -0.667 | -0.3527 | Yes |
| 39 | SPCS3 |  |  | 4512 | -0.686 | -0.3387 | Yes |
| 40 | XPOT |  |  | 4515 | -0.687 | -0.3179 | Yes |
| 41 | SLC7A5 |  |  | 4544 | -0.701 | -0.3017 | Yes |
| 42 | EIF2S1 |  |  | 4553 | -0.707 | -0.2815 | Yes |
| 43 | NOP56 |  |  | 4556 | -0.708 | -0.2601 | Yes |
| 44 | DKC1 |  |  | 4646 | -0.763 | -0.2536 | Yes |
| 45 | SEC11A |  |  | 4647 | -0.763 | -0.2302 | Yes |
| 46 | CEBPB |  |  | 4835 | -0.902 | -0.2382 | Yes |
| 47 | PSAT1 |  |  | 4867 | -0.932 | -0.2154 | Yes |
| 48 | SHC1 |  |  | 4885 | -0.948 | -0.1895 | Yes |
| 49 | KDELR3 |  |  | 4912 | -0.981 | -0.1643 | Yes |
| 50 | DDX10 |  |  | 5008 | -1.109 | -0.1484 | Yes |
| 51 | XBP1 |  |  | 5032 | -1.162 | -0.1170 | Yes |
| 52 | NPM1 |  |  | 5048 | -1.184 | -0.0835 | Yes |
| 53 | MTHFD2 |  |  | 5101 | -1.297 | -0.0535 | Yes |
| 54 | CKS1B |  |  | 5121 | -1.338 | -0.0160 | Yes |
| 55 | NABP1 |  |  | 5180 | -1.563 | 0.0210 | Yes |
Table: GSEA details [plain text format]

  

Fig 2: HALLMARK\_UNFOLDED\_PROTEIN\_RESPONSE: Random ES distribution      
 Gene set null distribution of ES for **HALLMARK\_UNFOLDED\_PROTEIN\_RESPONSE**
